# Disparities among 2009 Pandemic Influenza A (H1N1) Hospital Admissions: A Mixed Methods Analysis – Illinois, April–December 2009

**DOI:** 10.1371/journal.pone.0084380

**Published:** 2014-04-28

**Authors:** Kenneth Soyemi, Andrew Medina-Marino, Ronda Sinkowitz-Cochran, Amy Schneider, Rashid Njai, Marian McDonald, Maleeka Glover, Jocelyn Garcia, Allison E. Aiello

**Affiliations:** 1 Office of Health Protection, Division of Infectious Diseases, Illinois Department of Public Health, Chicago, Illinois, United States of America; 2 Epidemic Intelligence Service, Office of Surveillance, Epidemiology, and Laboratory Services, Centers for Disease Control and Prevention, Atlanta, Georgia, United States of America; 3 Division of Healthcare Quality Promotion, National Center for Emerging and Zoonotic Infectious Diseases, Centers for Disease Control and Prevention, Atlanta, Georgia, United States of America; 4 Office of Non communicable Diseases, Injury and Environmental Health, National Center for Chronic Disease Prevention and Health Promotion, Centers for Disease Control and Prevention, Atlanta, Georgia, United States of America; 5 Office of Health Disparities, National Center for Emerging and Zoonotic Infectious Diseases, Centers for Disease Control and Prevention, Atlanta, Georgia, United States of America; 6 Office of Director, Center for Infectious Diseases, Centers for Disease Control and Prevention, Atlanta, Georgia, United States of America; 7 University of Miami, Miller School of Medicine, Obstetrics and Gynecology Department, Miami, Florida, United States of America; 8 University of Michigan, School of Public Health, Ann Arbor, Michigan, United States of America; Harvard School of Public Health, United States of America

## Abstract

During late April 2009, the first cases of 2009 pandemic influenza A (H1N1) (pH1N1) in Illinois were reported. On-going, sustained local transmission resulted in an estimated 500,000 infected persons. We conducted a mixed method analysis using both quantitative (surveillance) and qualitative (interview) data; surveillance data was used to analyze demographic distribution of hospitalized cases and follow-up interview data was used to assess health seeking behavior. Invitations to participate in a telephone interview were sent to 120 randomly selected Illinois residents that were hospitalized during April–December 2009. During April–December 2009, 2,824 pH1N1 hospitalizations occurred in Illinois hospitals; median age (interquartile range) at admission was 24 (range: 6–49) years. Hospitalization rates/100,000 persons for blacks and Hispanics, regardless of age or sex were 2–3 times greater than for whites (blacks, 36/100,000 (95% Confidence Interval ([95% CI], 33–39)); Hispanics, 35/100,000 [95%CI,32–37] (; whites, 13/100,000[95%CI, 12–14); *p*<0.001). Mortality rates were higher for blacks (0.9/100,000; p<0.09) and Hispanics (1/100,000; p<0.04) when compared with the mortality rates for whites (0.6/100,000). Of 33 interview respondents, 31 (94%) stated that they had heard of pH1N1 before being hospitalized, and 24 (73%) did not believed they were at risk for pH1N1. On average, respondents reported experiencing symptoms for 2 days (range: 1–7) before seeking medical care. When asked how to prevent pH1N1 infection in the future, the most common responses were getting vaccinated and practicing hand hygiene. Blacks and Hispanics in Illinois experienced disproportionate pH1N1 hospitalization and mortality rates. Public health education and outreach efforts in preparation for future influenza pandemics should include prevention messaging focused on perception of risk, and ensure community wide access to prevention messages and practices.

## Introduction

During April 2009, the World Health Organization (WHO) declared a “public health emergency of international concern” in response to the first cases of infection with pandemic 2009 influenza A (H1N1) (pH1N1) in California and Mexico [1]. By early June 2009, WHO declared a pandemic [2]. The first case of pH1N1 infection in Illinois was reported April 25, 2009. Laboratory-based surveillance data demonstrated that pH1N1 virus became the predominant circulating influenza virus in Illinois within 2 weeks of its detection [3]. The novel virus, which contain segments of avian, human, and swine origins, typically caused mild disease [4] and it is estimated that on-going, sustained local transmission resulted in an estimated 500,000 infected persons [5].

Illinois was affected during both the spring (April–June 2009) and fall (August–December 2009) pandemic waves. To better understand the epidemiologic and clinical features of this pandemic, the Illinois Department of Public Health (IDPH) initiated enhanced surveillance for pH1N1 infection on April 25, 2009 by using the Illinois National Electronic Data Surveillance System to collect clinical case data for hospitalizations and fatal cases.

Study objectives were to 1) describe demographic and basic clinical characteristics of persons hospitalized with pH1N1 and 2) identify individual-level factors regarding health seeking behavior amongst those hospitalized with pH1N1.

## Methods

### Quantitative (Surveillance) Data

Illinois National Electronic Data Surveillance System (I-NEDDS) data was entered by hospital infection control practitioners and local health department staff. Data regarding Illinois residents hospitalized with laboratory-confirmed pH1N1 during April–December 2009 was extracted from I-NEDSS. A hospital admission was defined as an overnight stay in an Illinois hospital by an Illinois resident regardless of age. Laboratory confirmation was defined as a positive result using any of the following diagnostic measures: reverse-transcriptase polymerase chain reaction (RT-PCR) test for the pH1N1 virus; an influenza A RT-PCR test that was negative for human H1 and H3; a rapid influenza A antigen test; direct immunofluorescence assay; or viral culture. Each hospitalized patient was assigned a unique identifier. Race and ethnicity were recorded as reported by hospitalized patients in I-NEDSS; mortality was defined as a death during April–December 2009 in a person in which pH1N1 was the leading or a contributing cause of death.

High-risk medical co-morbidities, as defined by the Advisory Committee on Immunization Practices, included chronic pulmonary (including asthma), cardiovascular (excluding hypertension), renal, hepatic, neurologic/neuromuscular, hematologic, and metabolic disorders (including diabetes mellitus); immune suppression, including immune suppression caused by medications or human immunodeficiency virus; and pregnancy, including up to 6 weeks postpartum [6].

### Qualitative (Interview) Data

One hundred twenty randomly selected patients who had been hospitalized with pH1N1 during April 23, 2009–December 31, 2009 were mailed invitations to participate in telephone interviews designed to investigate health seeking behavior related to their hospitalization. Patients were stratified by IDPH immunization region and race/ethnicity; patients who were not Illinois residents were excluded. After stratification, patients were selected by random sampling from each of the strata; the number of patients selected from each stratum was proportional to representation in the state. To improve representation of minorities in the interview, non-Hispanic blacks and Hispanic patients were oversampled. Letters of introduction were followed by telephone contact approximately one week later. At least 2 telephone attempts were made to contact all patients. All interviews were conducted during April–June 2010.

Upon contact, oral consent to be interviewed was obtained. In the event that the hospitalized individual was a minor child, a parent was interviewed. The confidentiality of the respondents and their responses was assured at the beginning of the interview. All interviewers were trained to use a standardized script. The interview included both closed- and open-ended questions. Standardized probes were used to investigate symptoms, vaccination history, treatment history, access-to-health-care, insurance coverage, presence or absence of primary care physicians, personal perceptions, and cues to action for preventing influenza illness in the future. The interviews were transcribed in real time by a 3–5 person interview team. If the interviewee only spoke Spanish, a bi-lingual interview team re-contacted the individual and the interview was conducted in Spanish. After all interviews were completed, the transcripts were qualitatively coded for themes by using an immersion and crystallization process and aggregated into categories for analysis.

This investigation was conducted as part of public health practice and was classified as non-research by the Chicago Department of Public Health institutional review board and a human subjects review coordinator at the Centers for Disease Control and Prevention.

### Statistical Analysis

Rates per 100,000 population were calculated by using the 2009 midyear population estimates published by the Census Bureau as denominators [7]; rates were stratified by sex, race/ethnicity, and age group. Rates were suppressed when the numerator count was <5. Differences in proportions were evaluated using Pearson chi-square or Fisher's exact test. All reported *p* values were 2-sided, and a *p* value of <.05 was considered a statistically significant. Quantitative analysis was completed using SAS®, version 9.13 (SAS Institute, Inc., Cary, North Carolina).

## Results

### Quantitative (Surveillance) Analysis

**Figure 1:**
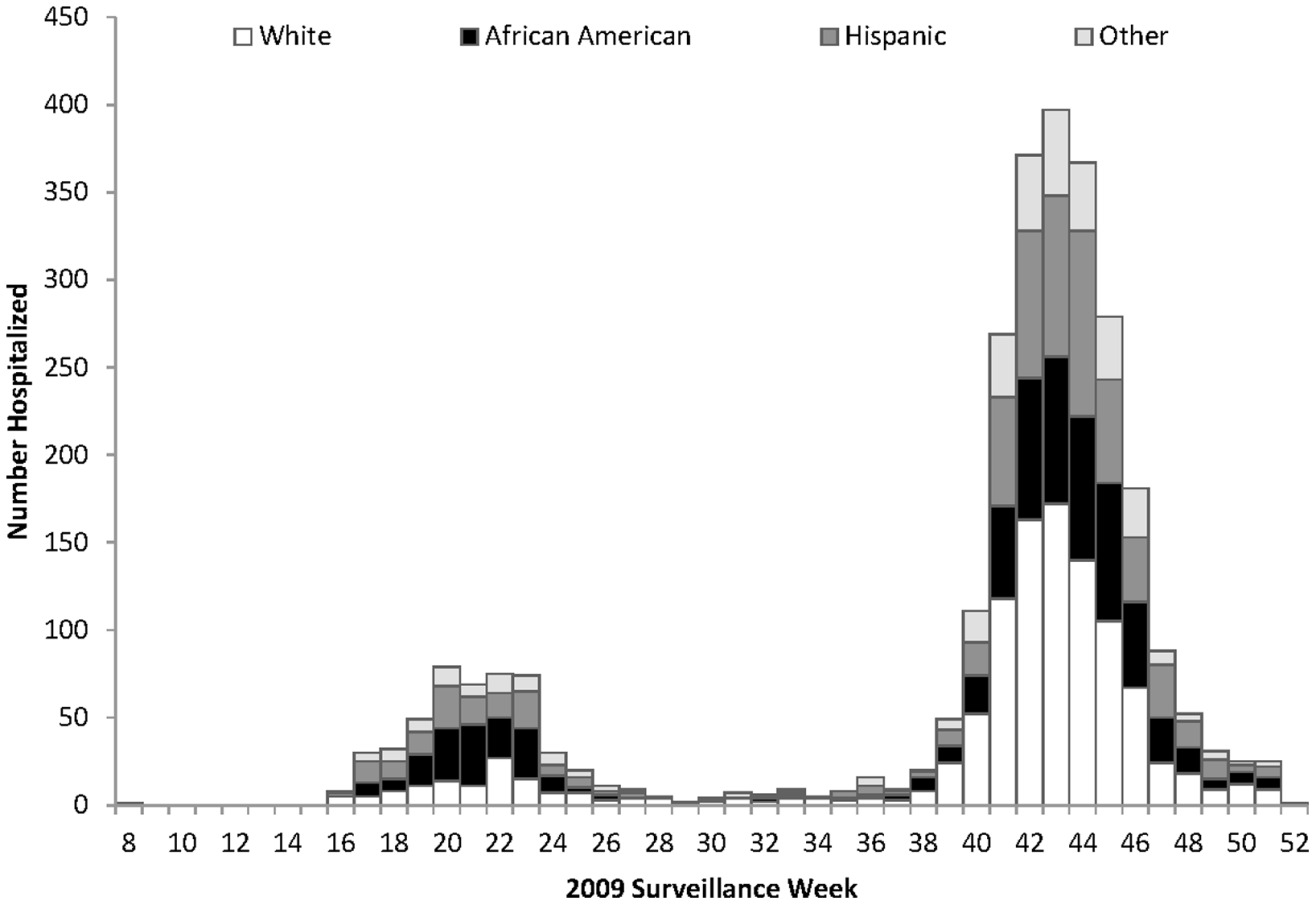
Data of pandemic 2009 influenza A (H1N1) hospitalizations by race and ethnicity, Illinois April –December 2009.

A total of 2,824 pH1N1 hospital admissions were identified in Illinois during April–December 2009 (Table 1). Hospital admissions peaked during surveillance week 20 (May 2009) in the first wave of the pandemic, and during surveillance week 43 (November 2009) in the second wave (Figure 1). Of the 2,824 identified admissions, 1,333 patients (47%) were male, median (interquartile range [IQR]) age at admission was 23.7 years (range: 6–49). Among the 2,824 admissions, non-Hispanic whites accounted for 1,068 (38%); blacks, 707 (25%); Hispanics, 680 (24%); Asian/Pacific Islanders, 107 (4%); Native Americans, 6 (0.2%); other, 63 (2%); and 193 (7%) were unknown. The median (IQR) hospital length of stay (HLOS) was 2 (range: 1–4) days. Of 7,651 total inpatient days, whites accounted for 3,500 (46%) days; blacks, 1,648 (22%) days; and Hispanics, 1,560 (20%) days. Residents aged <50 years accounted for 5,394 (71%) inpatient days. Approximately half of the patients (1,439 [51%]) were admitted into teaching hospitals, and 1,231 (44%) into community hospitals. Information on hospital type was unknown for 154 (5%) of hospital admissions. Of hospitalized patients, 324 (11%) reported ≥1 medical co-morbidity; of these, ≥2 medical co-morbidities were reported by 65 (20%) patients. The prevalence of co-morbidities was higher for blacks (126 [18%] of 707) and Hispanics (80 [12%] of 680), compared with that of whites (76 [7%] of 1,068). Compared with a hospitalization rate of 13/100,000 (95% Confidence Interval [95%CI] 12–14) population for whites, the hospitalization rate for blacks was 36 admissions/100,000 population [95%CI, 33–39] (rate ratio, 3; *p*<0.001) and for Hispanics was 35 admissions/100,000 population [95% CI, 32–37] (rate ratio, 2.6; *p*<0.001). There were 91 deaths, resulting in a case fatality rate of 3%. Of these 91 deaths, 47 (52%) were whites, 19 (21%) were Hispanics, 17 (19%) were blacks, 6 (7%) were unknown, 1(1%) was Asian and 1(1%) was classified as other. Mortality rates were higher for blacks (0.9/100,000; *p*<0.09) and Hispanics (1/100,000; *p*<0.04) than whites (0.6/100,000) (Table 2). Regardless of sex, hospitalization rates for blacks and Hispanics, were significantly higher compared to whites (data not shown).

**Table 1:** Demographics of residents hospitalized with 2009 influenza (A) H1N1 in Illinois, April–December, 2009.

Characteristic	Value
No. of residents hospitalized	2,824
Male, no. (%)^a^	1,333 (47)
Median age, yrs (IQR)^b^	23.7 (6–49)
Admitted to ICU^c^, no. (%)	421 (15)
Reported ≥1medical co morbidity	324 (11)
Co morbidity prevalence, (number with ≥1 co morbidity/total admitted [%])	
White	76/1,068 (7)
Black	126/707 (18)
Asian/Native Hawaiian/Pacific Islander	8/107 (7)
Native America/American Indian	0/6 (0)
Hispanic	80/680 (12)
Other	6/63 (10)
Unknown	28/193 (15)
Received antiviral therapy, no. (%)	1,688 (60)
Median hospital length of stay^c^, days, (IQR)^b^	2 (1–4)
Total inpatient days	7,651
Pregnant, no. (%)	110 (3.9)
Age Group	
<1 year, no. (%)	257 (9)
1–4 yrs, no. (%)	322 (11)
5–24 yrs, no. (%)	885 (31)
25–49 yrs, no. (%)	706 (25)
50–64 yrs, no. (%)	470 (17)
≥65 yrs, no. (%)	184 (7)
Hospital type	
Teaching hospital, no. (%)	1,439 (51)
Community hospital, no. (%)	1,231(44)
Unknown, No. (%)	154 (5)

a Percentages might total more than 100 because of rounding.

b Interquartile range.

c Intensive care unit.

**Table 2:** Hospital admission data for 2009 influenza A (H1N1) by race/ethnicity, Illinois, April–December 2009.

Race/Ethnicity	Number no. (%)^a^	Hospital Admission rate^b^	Inpatient days No. (%)^a^	MedianHLOS^c^ days, (IQR)^d^	ICU^e^ Admission No.(%)^f^	Number admitted to ICU^e^ reporting ≥1 medical co morbidity no.(%)^g^	Deaths No. (%)^a^	Mortality rate^b^
White	1,068 (38)	12.7	3,500 (46)	2 (2–4)	187 (18)	16 (9)	47 (52)	0.6
Black	707 (25)	33.2	1,648 (22)	2 (1–4)	101 (14)	24 (24)	17 (19)	0.9
Asian/Native Hawaiian/PI^h^	107 (4)	19.0	312 (4)	2 (1–4)	11 (10)	0 (0)	1 (1)	**
Native America/American Indian	6 (0)	25.8	22 (0.1)	5 (1–11)	1 (17)	0 (0)	0	0
Hispanic	680 (24)	31.5	1,560 (20)	2 (1–3)	85 (13)	13 (15)	19 (21)	1.0
Other	63 (2)	-	145 (2)	2 (1–4)	10 (16)	1(10)	1 (1)	**
Unknown	193 (7)	-	464 (6)	3 (1–4)	26 (13)	4 (15)	6 (7)	-
Total	2,824 (100)	21.8	7,651(100)	2 (1–4)	421(15)	58 (14)	91 (100)	1.0

a Percentages might total more than 100 because of rounding.

b Rate per 100,000 population.

c Hospital Length of stay.

d Interquartile range.

e Intensive care unit.

f Denominator is the number of admissions (column 2).

g Denominator is the number of ICU admissions (column 5).

h Pacific Islander.

**Rates suppressed because of low cell count.

State-wide hospitalization rates were inversely proportional to age group, with hospital admission rates highest for children aged <5 years and lowest for residents aged >65 years. For all age groups, hospitalization rates were higher for blacks and Hispanics than for whites (*p*<0.001). For persons aged ≥65 years, the hospitalization rate for Hispanics (40 per 100,000 population; [95% CI, 28–56]) was substantially greater than that for whites (8 per 100,000[95% CI, 6–9) or blacks (18 per 100,000[95%CI, 12–25) *p*<0.001) (Table 3).When stratified by IDPH immunization regions, the cumulative hospitalization rates of varied by region, with the Rockford (35 per 100,000 [95%CI, 31–40]) and Chicago Cook (29 per 100,000[95%CI, 27–30]) regions reported the highest rates and Edwardsville reported the lowest 7.3 per 100,000[95%CI, 6–9]).Similarly, for all racial and ethnic groups, Rockford region had the highest hospital admission rates.

**Table 3:** 2009 influenza A(H1N1) hospital admission rates/100,000 population by race/ethnicity and age group, Illinois, April–December 2009.

	Race/Ethnicity				
Age group	White^a^	Black	Hispanic	Statewide	*P* value^b^
0–4 yrs Rates (95% C.I.)^c^	30 (25–35)	91(76–109)	90 (78–103)	61(56–66)	<0.001
5–24 yrs Rates (95% C.I.)	15 (14–17)	40 (35–46)	30 (26–35)	25 (24–27)	<0.001
25–64 yrs Rates (95% C.I.)	12 (11–13)	32 (28–36)	24 (21–27)	18 (17–19)	<0.001
≥65 yrs Rates (95% C.I.)	8 (6–9)	18 (12–25)	40 (28–56)	12 (10–14)	<0.001

a Referent group.

b *P* value calculated chi-square test (Fisher's exact or Pearson).

c 95% Confidence Interval.

Severe clinical presentation requiring intensive care unit (ICU) admission was reported by 421 of 2,824 (15%) patients. The proportion of ICU to total admissions was higher for whites; however, the proportion of patients admitted into the ICU who reported ≥1 medical co-morbidity was lower for whites than for other racial groups.

### Qualitative (Interview) Analysis

Of the 120 Illinois residents randomly selected to participate in the interview, phone contact was established with 60, of which 33 (55%) completed the interview. Among respondents, 13 (39%) were white; 10 (30%) were black; and 10 (30%) were Hispanic; the racial and ethnic distribution was similar to the overall random sample (Table 1). One respondent was admitted to the hospital during the first wave (Table 4). The median (IQR) age of respondents was 31 years (range: 8–53); 19 (58%) respondents were male.

**Table 4:** Qualitative demographical, clinical, and qualitative interview responses among 33 Illinois residents hospitalized with 2009 influenza A (H1N1) — April–December, 2009.

Characteristic	Frequency No. (%)
Response rate	33/60 (55)
White	13/33 (39)
Male	19/33(58)
Admitted during 1st wave	1/33 (3)
Aged <18 years	11/33 (33)
Admitted to ICU^a^	3/33 (9)
Mechanical ventilation	0/33 (0)
Antiviral therapy	21/33 (64)
Heard of H1N1 before hospitalization	31/33 (94)
Did not feel at risk for getting H1N1	24/33 (73)
Did not receive H1N1 vaccination	27/29 (93)
Lack of availability	15/27 (56)
Just did not	4/27 (15)
Side-effects	2/27 (7)
Doctor advice	1/27 (4)
Too young	1/27 (4)
Reaction to vaccine	1/27 (4)
Got sick before getting vaccine	1/27 (4)
Injectable form not available	1/27 (4)
Do not get vaccinations	1/27 (4)
Missed chance at work because of illness	1/27 (4)
Had health insurance at time of admission	29/31 (94)
Did not seek healthcare before admission	19/32 (59)
Rapid onset of symptoms	12/33 (36)
Used OTC^b^ medications	15/33 (45)
Presence of health-seeking barrier	5/33 (15)

a Intensive care unit.

b Over-the-counter.

After becoming sick, 15 (45%) respondents reported the use of over-the-counter medication as a method of treatment. Other responses included use of an inhaler or nebulizer, consulting with a personal physician, use of antibiotics, drinking fluids, use of home remedies, resting, taking a bath, use of a prescription or Tamiflu® (Genentech, Inc., South San Francisco, California), and seeking medical care at the hospital. Eight respondents reported that they did not treat symptoms before seeking medical care at the hospital.

Nineteen respondents (58%) stated that they did not pursue any other medical evaluation before seeking medical care at the hospital. Eight (24%) respondents reported specific barriers that delayed their decision to seek healthcare. The reported barriers (number reporting them) are as follows: cost of care or lack of healthcare coverage (1), no paid sick leave (1), missing work (1), transportation (1), language or communication barrier (1), dislike of hospitals and overcrowded emergency departments (1), no ideal treatment (1), and not wanting to find a new doctor (1).

Of the 8 participants that reported a barrier the average time from symptom onset to hospital admission was 2.69 days. Of those that reported no barriers to seeking healthcare (n = 23), the average time from symptom onset to hospital admission was 1.89 days.

The median interval between onset of symptoms and seeking initial treatment from a healthcare provider was 2 (range: 1–7) days; this interval was similar for all racial/ethnic groups. The median interval between onset of symptoms and hospital admission was 4 days for both blacks and for Hispanics, compared with 2 days for whites. When stratified by age group, race and ethnicity there were differences in longest mean of interval reported. For Whites, respondents aged between 5 and 24 years reported the longest mean duration (4.3 days), Black respondents aged 50–64 years reported longest mean duration of 6.5 days, and Hispanics respondents aged 65 years and above reported the longest mean duration (6.0 days).

The median (IQR) HLOS of the respondents was 2 days (range: 2–4.5); overall the mean HLOS was highest for blacks (3.8 days) compared with White (3.0 days), and Hispanic (2.8 days). When HLOS was stratified by age group and race, individuals aged 50–64 years for all race and ethnic groups reported the longest mean HLOS. Three (9%) of the respondents were admitted into the ICU.

Among respondents, 31 (94%) stated that they had heard of pH1N1 (also referred to as swine flu) before hospitalization (Table 4). For each racial and ethnic group there were variations in the proportions of age groups who had heard about H1N1. Of those who had heard these terms, the news media was cited as being the most common source (77%), followed by primary healthcare provider (10%), workplace (10%), and from family or friends (6%).

When asked about perception of risk, 24 (73%) stated that they did not perceive themselves to be at risk for acquiring H1N1 before becoming sick (Table 4) because they were either not around sick people or they rarely got sick. The proportion of respondents who perceived themselves to be at risk was greater for whites (6 [46%] of 13) than for blacks (2 [20%] of 10) or Hispanics (1 [10%] of 10).

The main reason cited by respondents for not receiving the vaccine was lack of availability. Of the 33 respondents, 29 were hospitalized after mid October 2009 when the monovalent H1N1 vaccine became available; of these, 27 (93%) responded that they did not receive the vaccine (Table 4). Twenty-nine (88%) reported having some form of health insurance coverage. When asked about usual source of healthcare, 27 (82%) respondents stated that they most often go to their personal healthcare provider, 5 (15%) said the emergency department, and 1 (3%) said a community health clinic.

When asked how to prevent pH1N1 infection in the future, the two most common responses were 1) get vaccinated and 2) practice hand hygiene, both indicated by 15 (28%) of 53 total responses received. One participant stated, “Make sure that everybody had the shot. It almost took my life, and I can imagine what it can do to somebody else's.” At the conclusion of the interview, respondents were asked what they wish they had known or what single piece of advice they would give to someone else on the basis of their experiences with pH1N1. Ten of 46 (22%) responses focused on getting vaccinated, 5 of 46 (11%) focused on seeking medical attention, and practicing hand hygiene, staying away from sick people, and educating yourself and others were each mentioned 4 of 46 (9%) times.

## Discussion

This study was a mixed methods analysis, using quantitative (surveillance) hospital admission data and qualitative (interview) data obtained from follow-up interviews of a sample of Illinois residents who were hospitalized during April–December 2009. Consistent with other studies [8]–[15], pH1N1 hospitalization and mortality rates among blacks and Hispanics in Illinois were higher than those for whites.

It is unclear whether the disparities in pH1N1 hospitalization and mortality rates documented in our study and others are attributable to differences in disease incidence, disparities of underlying chronic conditions, or unequal access to healthcare leading to differences in timely care-seeking behaviors [16]. Factors that have contributed to disparities in influenza incidence among minorities during previous influenza seasons include lower rates of vaccination coverage resulting in islands of susceptibility, and differences in viral circulation among geographic regions [17]–[21]. Previous studies have also attributed the increased risk for complications and mortality among minorities to the higher burden of chronic diseases within these populations. In our study, the prevalence of co-morbidities among patients hospitalized with pH1N1 in Illinois was higher for blacks and Hispanics than for whites. It has also been proposed that unequal access to healthcare by minorities may lead to delays in treatment, thus contributing to disparities in morbidity and mortality [9], [22]. The small sample size of our interview respondents precludes statistical comparison however, although the time from symptom onset to seeking initial healthcare was similar among racial/ethnic categories, the median interval between onset of symptoms and hospitalization was longer for blacks and Hispanics (4 days) than for whites (2 days).

Our finding that state-wide hospitalization rates were inversely proportional to age group was similar to that of California [23]. The median age of Illinois residents hospitalized with pH1N1 was 24 years, similar to the median age of 25 years in California [23]. Reasons for the relative decrease in hospitalization rates for older persons with pH1N1 are unclear. Though studies have confirmed that pre-existing antibodies may have protected older persons from pH1N1 infection, diminished cell-mediated immunity might have contributed to greater severity after infection [24]. Though hospitalization rates decreased with increasing age for whites and blacks, this was not the case for Hispanics. Our study documented an admission rate for Hispanics >65 years old that was substantially higher than those for whites or blacks, and there is no epidemiologic or clinical evidence suggesting that older Hispanics in Illinois are more susceptible to pH1N1 by virtue of ethnicity alone.

Policy makers and public health officials should develop and promote culturally sensitive communication and health education campaigns to improve vaccination rates and other methods of prevention (e.g., social distancing, hand washing) among minorities and hard to reach populations. Because of the higher hospitalization rates among patients aged <5 years, public health authorities should improve access to vaccines, and employers should explore ways to improve sick-leave and telework policies for parents of ill children.

The low receipt of the pH1N1 vaccine among survey respondents in our study is similar to results from the Behavioral Risk Factor Surveillance System and the National 2009 H1N1 Flu Survey. Specifically, these results demonstrated vaccine uptake estimates in Illinois of 25.1% for all residents, 21.6% of residents aged ≥18 years, and 37.5% of residents aged 6 months–17 years [25]. When asked why they had not received the flu vaccine, approximately half of our respondents cited non-availability. Surveys in the United States before the availability of the pH1N1vaccine reported that 40%–60% of U.S. adults intended to receive the H1N1 vaccine when it became available [26]–[28]. Vaccination intentions for pH1N1 were strongly associated with seasonal influenza vaccinations, indicating common attitudinal barriers to both vaccines [26]. Assuming a constant vaccine supply, future studies should explore the relationship between a willingness to receive the vaccine, and actually obtaining it.

Severe disease from pH1N1 infection has been associated with a longer interval from onset of symptoms to treatment with antiviral therapy, and with the presence of underlying co-morbidities [29]–[32] In our study, interview respondents reported delays (median, 2 days) between onset of symptoms and decision to seek care; this did not change when adjusted for ICU admission. Of 33 respondents, 12 (36%) reported that rapidity of symptom onset was the reason for seeking medical care. A majority of respondents did not report inadequate access to care, lack of a medical home, or lack of insurance as barriers to receiving healthcare.

Certain respondents agreed that vaccination, early medical care, hand hygiene, and social distancing were important preventive methods for the future.

Knowledge has a considerable influence on attitudes and practices during a pandemic, and personal experience influences behaviors. A person's response to the threat of disease is dependent on perception of risk, which itself is influenced by public and private information disseminated by the media [33]. Although 31of the 33 (93%) interview respondents had heard about pH1N1 in the news, indicating the importance of mass media as an information source, 24 of the 33 respondents (72%) felt they were not at risk of being infected. The relatively low level of mortality reported during this pandemic and its influence on risk perception and vaccine seeking behavior [34], should be evaluated. When levels of worry are low, acting to increase the volume of mass media and advertising coverage is likely to increase the perceived efficacy of recommended behaviors and in turn likely to increase the uptake of recommended preventive measures [35]. Shortcomings in educational outreach might have been a factor in our respondents' understanding of their risk for infection. Therefore, efforts should be targeted at educating the general population to improve practices for future epidemics [36].

Racial/ethnic minority populations have higher hospitalization and mortality rates, and poorer health outcomes for pH1N1, and other diseases, than do whites [15], [18], [37]–[39]. Pandemic preparedness and response can be improved by 1) developing culturally and linguistically sensitive communication plans that address the specific needs of minority communities, 2) enhancing health system safety nets, and 3) developing social policies that minimize economic burdens and improve compliance with isolation and quarantine, [40].

One limitation of our study was that we did not validate I-NEDSS surveillance data regarding hospitalizations or co-morbidities with medical records abstraction. As a result, we were unable to assess factors relating to insurance type, education level or household size; such information may have informed us about the influence of socioeconomic circumstances on health. Also, because height and weight information were not available, we could not assess the association between obesity and severity of illness that has been established in previous studies. Regarding co-morbidities, previous studies have reported that 62–73% of hospitalized patients presented with an underlying medical condition [23], [41], [42]. Our reporting that 11% of hospitalized patients had underlying medical conditions likely reflects an underreporting of co-morbidities into I-NEDSS.

False-negative test results, lack of testing due to a low clinical index of disease suspicion, and the belief that testing was not essentially for management of patients may have contributed to an undercounting of the true burden of pH1N1 in hospitalized patients. Because interviews were conducted months after hospitalization, study respondents might not have recalled details of events surrounding hospitalization. Finally, interviews were conducted among a sample of only 33 Illinois residents hospitalized for pH1N1. Consequently, our study was underpowered to assess differences among racial/ethnic groups, and our findings might not be representative of all pH1N1-infected residents who were hospitalized. Despite these limitations, these data provide insight into perceptions of patients infected and hospitalized with pH1N1.

Future pandemic mitigation strategies should include communication methods that address risk perception (targeted to hard-to-reach communities, including minorities). We recommend preventive measures that emphasize the provision and early access to antiviral medications and vaccines [1], [43], [44].
